# Paraumbilical vein aneurysm: case report

**DOI:** 10.1259/bjrcr.20190052

**Published:** 2020-02-12

**Authors:** Natasha Gardiner, Stephan Voigt

**Affiliations:** 1 Radiology Registrar, Queen Alexandra Hospital, Portsmouth, England; 2 Consultant Radiologist, St Mary’s Hospital, Isle of Wight, United Kingdom

## Abstract

A 48-year-old female patient was found to have a paraumbilical vein saccular aneurysm, which is a rare consequence of portal hypertension. She presented with right upper quadrant pain and had a known diagnosis of alcoholic liver disease. This had progressed since her last admission. We discuss the multimodality images obtained, diagnosis and complications associated with this pathology.

## Clinical presentation

A 48-year-old female patient with a known alcoholic liver disease history, presented to St Mary's Hospital, Isle of Wight with right upper quadrant pain. Her liver function tests were deranged, indicating an obstructive picture. Her amylase was normal.

## Investigations

**Figure 1.**
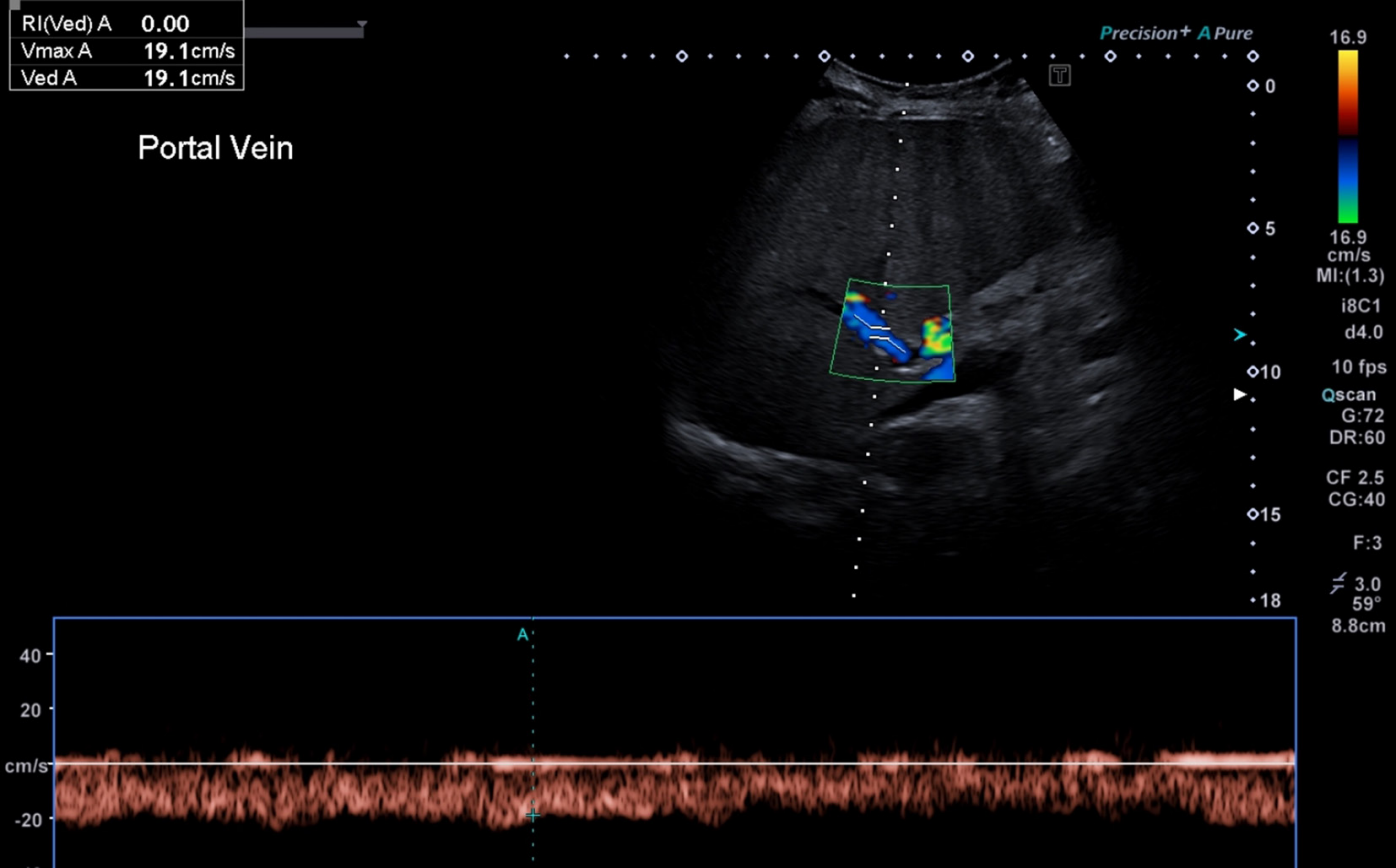
Ultrasound image showing retrograde flow in the portal vein, in keeping with alcoholic liver disease.

An abdominal ultrasound on the day of her admission illustrated features in keeping with cholecystitis. The gallbladder wall was thickened and a single gallstone was seen within the gallbladder but there was no evidence of biliary tract dilatation. Retrograde flow of 19 cm/s (Figure 1) was demonstrated within the portal vein and the liver was noted to be of a coarse echotexture—findings in keeping with alcoholic liver disease. No liver lesion was seen.

**Figure 2.**
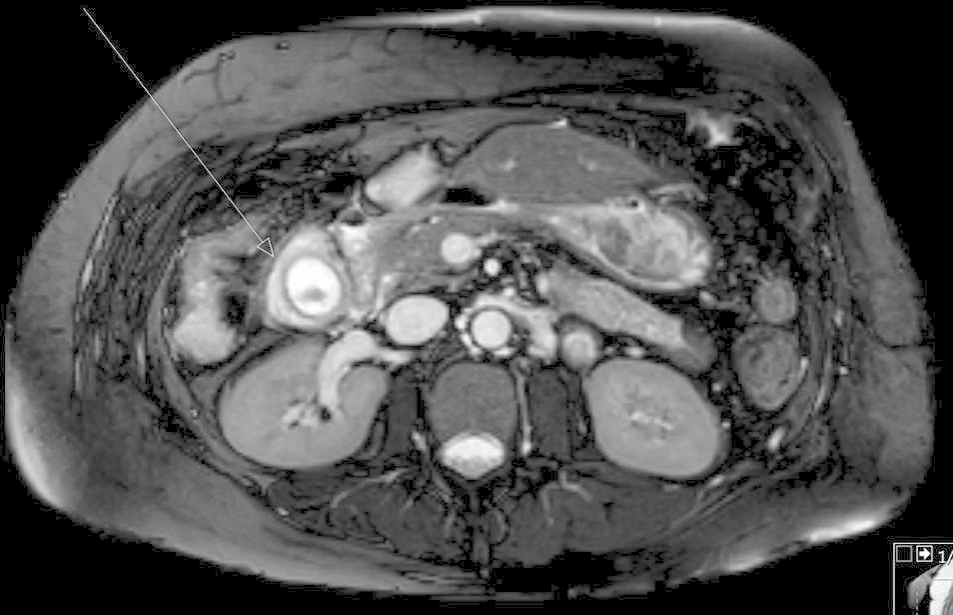
MR axial slice of upper abdomen showing pericholecystic thickening and a single low signal gallstone.

**Figure 3.**
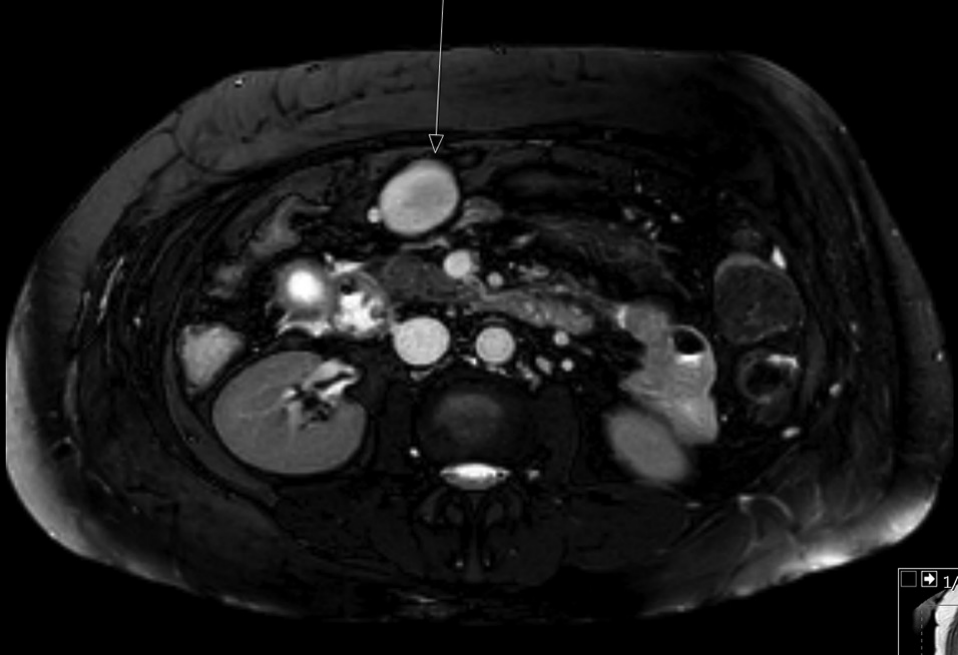
MR axial slice of upper abdomen showing high *T*_2_ signal ovoid structure.

Following this, a Philips Ingenia 1.5 T magnetic resonance cholangiopancreatography (MRCP) was requested to see if a small gallstone within the common bile duct or cystic duct was present and may have been missed on ultrasound. The MRCP found features of cholecystitis but no intraductal calculi. This feature are more clearly showed on this axial *T*_2_ image (*Figure 2*). The liver was noted to be cirrhotic and also multiple splenic varices were identified, indicating marked portal hypertension. The pancreas was unremarkable in appearance. However anterior to the pancreatic head and lying adjacent to vessels, a high *T*_2_ signal, well defined ovoid structure, measuring 40 × 28 mm was demonstrated (Figure 3).

## Differential diagnosis

- Pancreatic pseudocyst
- Pancreatic aneurysm
- Hepatic cirrhosis with varices
- Paraumbilical vein aneurysm

**Figure 4.**
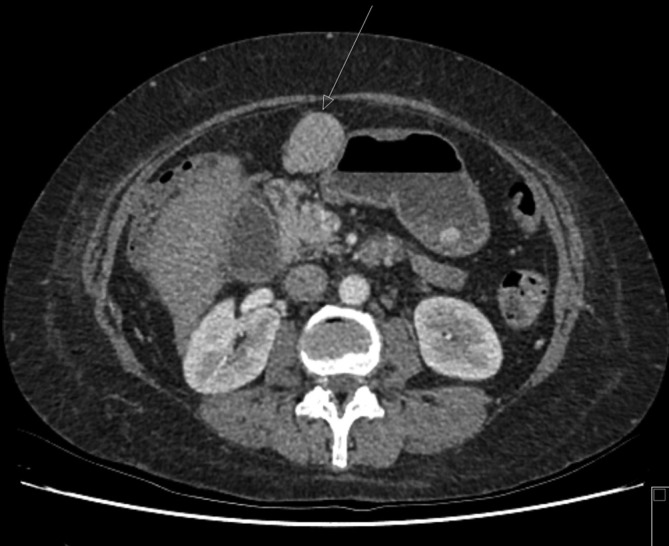
CT axial slice of upper abdomen showing a discrete ovoid structure, which fills with contrast and is seen to arise from the paraumbilical vein.

**Figure 5.**
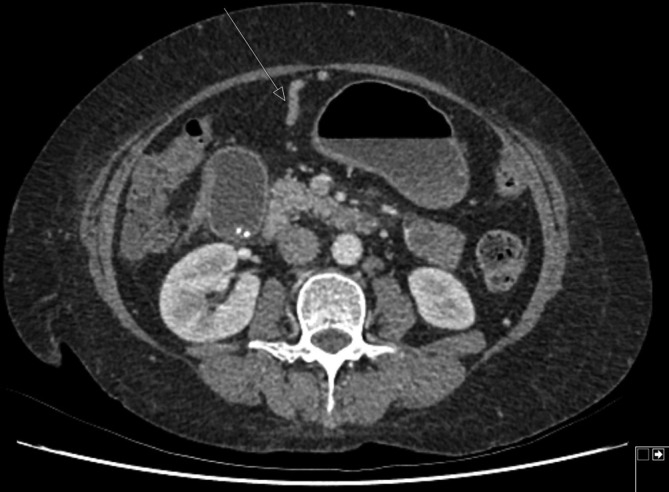
CT axial slice of upper abdomen showing a recanalised paraumbilical vein.

**Figure 6.**
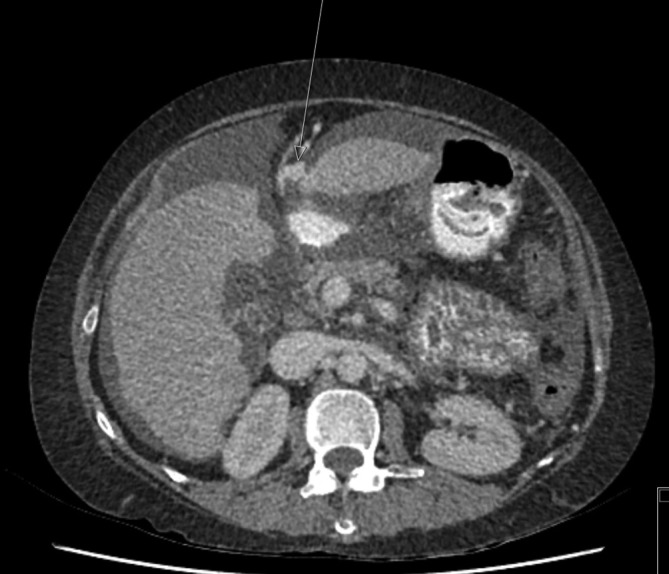
CT axial slice of the upper abdomen, from 2 years ago, showing a smaller paraumbilical vein aneurysm, arising from a dilated paraumbilical vein

A Siemens SOMATOM Definition AS 128 row CT pancreas was arranged to better assess the contents of the high *T*_2_ signal lesion and, more specifically, see if it was of vascular origin. CT is more accessible and has a shorter waiting time than MR in our institution. The dual phase contrast-enhanced CT scan of the upper abdomen showed a 38 × 30 mm lesion, which was anterior to the pancreas and discrete from the medial wall of the stomach and liver and segment D1 of the duodenum (Figure 4). The lesion was seen to arise from a recanalised paraumbilical vein (Figure 5), which is a branch of the left portal vein. A previous CT from 2 years earlier showed a smaller, 10 mm, lesion in a similar location arising from a dilated paraumbilical vein (Figure 6).

The above findings are consistent with this lesion representing a recanalized paraumbilical vein saccular aneurysm, which is increasing in size. No thrombus was demonstrated within the aneurysm.

## Discussion

The confluence of the superior mesenteric and splenic veins posterior to the pancreatic neck, give rise to the portal vein. The portal vein divides at the porta hepatis into the right and left portal veins. The right portal vein gives off branches to the caudate lobe and the right lobe of the liver. The left portal vein follows a horizontal course to the left and then turns medially towards the ligamentum teres and supplies liver segments II and III. Finally, it gives superior and inferior segmental branches to segment IV.^1^

The most common cause of portosystemic collateral vessels is portal hypertension.^1^ A study by Lafortune at al^2^ showed that in patients with portal hypertension it is the paraumbilical vessels, rather than the umbilical vessels, which increase in number and size. It has been suggested that the ligamentum teres, which surrounds the umbilical vein, may provide resistance to its dilatation. The paraumbilical veins connect the portal venous system to the systemic venous system and therefore provide a portosystemic shunt.

Portal hypertension is defined as elevation of the hepatic venous pressure gradient of >5 mmHg.^3^ It can be classified as pre-hepatic, hepatic or post-hepatic. Portal pressure, which can be measured in real time by ultrasound, is the gold-standard to evaluate the severity of portal hypertension.^4^ Imaging allows causes of high pressures to be sought. For example, portal vein thrombosis in prehepatic, cirrhosis in hepatic and thrombosis of the hepatic vein or inferior vena cava in posthepatic portal hypertension.^3^

Aneurysmal dilatation of a collateral vessel has been shown to be unusual^5,6^ in the progression of portal hypertension. Indeed, a specific sign of this is a patent paraumbilical vein demonstrated on duplex Doppler sonography.^7^ Portal hypertension more frequently presents as oesophageal varices, recurrent episodes of ascites, jaundice and hepatic encephalopathy.^8^

The progression of a paraumbilical vein aneurysm has not been outlined in previous reports.^6^ Note has been made of complications including thrombosis, rupture and obstructive jaundice. In cases of portal vein aneurysms, most need no treatment and follow up is sufficient. In acute cases, such as thrombosis, anticoagulation would be used for management.^9^

## Conclusion

This case provides a reminder that, although rare, a feature of portal hypertension is recanalization of a paraumbilical vein. Regular follow up for this pathology, rather than surgical treatment is recommended. However, if the aneurysm demonstrates a significant increase in size or complications, such as acute thrombosis, occur active treatment should be commenced.^6^

## Learning points

1. The left portal vein supplies liver segments II, III and gives superior and inferior segmental branches to segment IV.
2. The paraumbilical veins arise from the left portal vein and connect the portal venous system to the systemic venous system. A portosystemic shunt is provided.
3. A rare feature of portal hypertension, which is a direct consequence of alcoholic liver disease, is a recanalized paraumbilical vein aneurysm.

## References

[1] Gallego C, Velasco M, Marcuello P, Tejedor D, De Campo L, Friera A, et al Congenital and acquired anomalies of the portal venous system. Radiographics 2002; 22: 141–59. doi: 10.1148/radiographics.22.1.g02ja08141 11796904

[2] Lafortune M, Constantin A, Breton G, Légaré AG, Lavoie P The Recanalized umbilical vein in portal hypertension: a myth. AJR Am J Roentgenol 1985; 144: 549–53. doi: 10.2214/ajr.144.3.549 3881894

[3] Foster RJ, Cowell GW Acute paraumbilical vein recanalization: an unusual complication of acute pancreatitis. BJR|case reports 2015; 1: 20150021. doi: 10.1259/bjrcr.20150021 30363191 PMC6159162

[4] Maruyama H, Yokosuka O Ultrasonography for noninvasive assessment of portal hypertension. Gut Liver 2017; 11: 464–73. doi: 10.5009/gnl16078 28267700 PMC5491080

[5] Stuck KJ, Rubin JM, Gubin B Aneurysm of a paraumbilical collateral vein. Journal of Ultrasound Medicine 1988; 7: 639–42. doi: 10.7863/jum.1988.7.11.639 3210255

[6] Ohhira M, Matsumoto A, Ohhira M, Murazumi K, Ohta H, Ono M, et al Paraumbilical vein aneurysm. Case reports. Angiology 1996; 47: 517–21. doi: 10.1177/000331979604700512 8644950

[7] Gibson RN, Gibson PR, Donlan JD, Clunie DA Identification of a patent paraumbilical vein by using Doppler sonography: importance in the diagnosis of portal hypertension. AJR Am J Roentgenol 1989; 153: 513–6. doi: 10.2214/ajr.153.3.513 2669464

[8] Procopet B, Berzigotti A Diagnosis of cirrhosis and portal hypertension: imaging, non-invasive markers of fibrosis and liver biopsy. Gastroenterol Rep 2017; 5: 79–89. doi: 10.1093/gastro/gox012 PMC5421457 28533906

[9] Lee T-P, Lu HC, Chou Y-H, Tiu C-M, Chiou S-Y, Chiou H-J, et al Portal vein aneurysm: a case report and review of the literature. J Med Ultrasound 2009; 17: 57–62. doi: 10.1016/S0929-6441(09)60016-3

